# Healthcare utilisation in people with long COVID: an OpenSAFELY cohort study

**DOI:** 10.1186/s12916-024-03477-x

**Published:** 2024-06-20

**Authors:** Liang-Yu Lin, Alasdair D. Henderson, Oliver Carlile, Iain Dillingham, Ben F. C. Butler-Cole, Michael Marks, Andrew Briggs, Mark Jit, Laurie A. Tomlinson, Chris Bates, John Parry, Sebastian C. J. Bacon, Ben Goldacre, Amir Mehrkar, Brian MacKenna, The OpenSAFELY Collaborative, Rosalind M. Eggo, Emily Herrett

**Affiliations:** 1 Faculty of Epidemiology and Population Health, London School of Hygiene and Tropical Medicine, Keppel Street, London, WC1E 7HT UK; 2 Department of Primary Care Health Sciences, Bennett Institute for Applied Data Science, University of Oxford, NuffieldOxford, OX2 6GG UK; 3 TPP, TPP House, 129 Low Lane, Horsforth, Leeds, LS18 5PX UK; 4 Hospital for Tropical Diseases, University College London Hospital, London, WC1E 6JD UK; 5 Division of Infection and Immunity, University College London, London, London, WC1E 6BT UK; 6 Institute of Environmental and Occupational Health Sciences, National Taiwan University, Taipei, 100 Taiwan; 7 Department of Environmental and Occupational Medicine, National Taiwan University Hospital, Taipei, 100 Taiwan

**Keywords:** Long COVID, Electronic health records, Facilities and services utilization, Health care costs

## Abstract

**Background:**

Long COVID potentially increases healthcare utilisation and costs. However, its impact on the NHS remains to be determined.

**Methods:**

This study aims to assess the healthcare utilisation of individuals with long COVID. With the approval of NHS England, we conducted a matched cohort study using primary and secondary care data via OpenSAFELY, a platform for analysing anonymous electronic health records. The long COVID exposure group, defined by diagnostic codes, was matched with five comparators without long COVID between Nov 2020 and Jan 2023. We compared their total healthcare utilisation from GP consultations, prescriptions, hospital admissions, A&E visits, and outpatient appointments. Healthcare utilisation and costs were evaluated using a two-part model adjusting for covariates. Using a difference-in-difference model, we also compared healthcare utilisation after long COVID with pre-pandemic records.

**Results:**

We identified 52,988 individuals with a long COVID diagnosis, matched to 264,867 comparators without a diagnosis. In the 12 months post-diagnosis, there was strong evidence that those with long COVID were more likely to use healthcare resources (OR: 8.29, 95% CI: 7.74–8.87), and have 49% more healthcare utilisation (RR: 1.49, 95% CI: 1.48–1.51). Our model estimated that the long COVID group had 30 healthcare visits per year (predicted mean: 29.23, 95% CI: 28.58–29.92), compared to 16 in the comparator group (predicted mean visits: 16.04, 95% CI: 15.73–16.36). Individuals with long COVID were more likely to have non-zero healthcare expenditures (OR = 7.66, 95% CI = 7.20–8.15), with costs being 44% higher than the comparator group (cost ratio = 1.44, 95% CI: 1.39–1.50). The long COVID group costs approximately £2500 per person per year (predicted mean cost: £2562.50, 95% CI: £2335.60–£2819.22), and the comparator group costs £1500 (predicted mean cost: £1527.43, 95% CI: £1404.33–1664.45). Historically, individuals with long COVID utilised healthcare resources more frequently, but their average healthcare utilisation increased more after being diagnosed with long COVID, compared to the comparator group.

**Conclusions:**

Long COVID increases healthcare utilisation and costs. Public health policies should allocate more resources towards preventing, treating, and supporting individuals with long COVID.

**Supplementary Information:**

The online version contains supplementary material available at 10.1186/s12916-024-03477-x.

## Background

After infection with SARS-CoV-2, symptoms usually resolve in 4 weeks; however, for some people, the symptoms persist. The National Institute for Health and Care Excellence (NICE) defines symptoms lasting from 4 to 12 weeks as “ongoing symptomatic COVID-19” and longer than 12 weeks as “post-COVID-19 syndrome”. According to the NICE guidelines, ongoing symptomatic COVID-19 and post-COVID-19 syndrome both refer to long COVID. Common symptoms of long COVID include weakness, general malaise, fatigue, concentration impairment (known as “brain fog”), and breathlessness [1]. In March 2023, the Office for National Statistics reported that about 1.9 million people (approximately 2.9% of the UK population) had long COVID symptoms [2].

The persistent symptoms of long COVID affect quality of life [3], and patients seek care for their symptoms [4–6]. Evidence from the UK and other countries has demonstrated an increase in healthcare use and costs in groups with long COVID [7–9]. However, many of these studies define long COVID based on COVID-19 testing, which introduces selection bias due to testing policy, and reporting of testing [10–12].

There is an urgent need to fully quantify the healthcare use of patients with long COVID, to allow healthcare planning decisions and to properly quantify the impact of the COVID-19 pandemic on the healthcare system. Therefore, our study aims to investigate the healthcare utilisation of people with long COVID, factors associated with increased utilisation, and the associated cost to the NHS.

## Methods

### Data source

All data were linked, stored and analysed securely within the OpenSAFELY platform https://opensafely.org/. Data include pseudonymized data such as coded diagnoses, medications and physiological parameters. No free text data are included. All code is shared openly for review and re-use under the MIT open license (https://github.com/opensafely/openprompt_health_utilisation). Detailed pseudonymised patient data is potentially re-identifiable and therefore not shared. We rapidly delivered the OpenSAFELY data analysis platform without prior funding to deliver timely analyses on urgent research questions in the context of the global COVID-19 health emergency: now that the platform is established we are developing a formal process for external users to request access in collaboration with NHS England; details of this process are available at OpenSAFELY.org. Primary care records managed by the GP software provider, TPP, were linked to ONS death data, emergency care attendance, hospital admission, outpatient clinic visit records and costs data through OpenSAFELY. Derived from NHS England, OpenSAFELY-TPP data is representative of the English population [13].

### Study population and eligibility

**Fig. 1:**
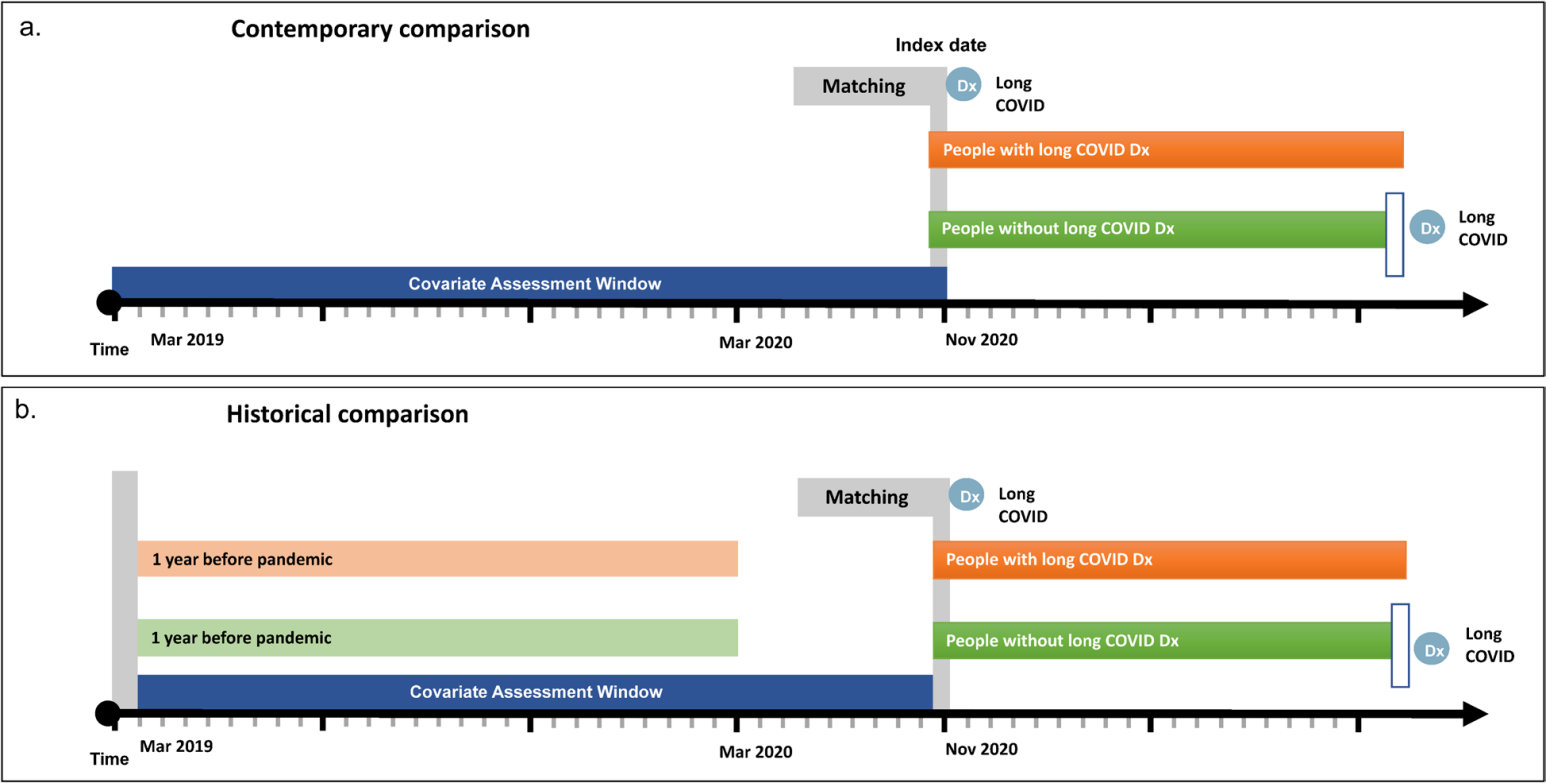
The study design. **a** Contemporary comparisons. **b** Historical comparisons

We conducted a matched retrospective cohort study using electronic health records and performed two comparisons: one contemporary and one historical (Fig. 1). A ‘contemporary’ comparison was designed to demonstrate differences in healthcare utilisation after long COVID diagnosis at a given calendar time compared to matched controls, when the pandemic or seasonal variation in illness might affect utilisation across the whole population. A ‘historical’ comparison was designed to understand differences between those with and without long COVID in terms of their previous healthcare utilisation and to examine the change in use within and between groups over time.

In the contemporary comparison, we followed the cohort from index date to the earliest of (1) date of death; (2) end of GP registration; (3) receipt of a resolved long COVID SNOMED code (1,326,351,000,000,108) among the exposed group; (4) receipt of a long COVID diagnosis among the unexposed group; and (5) 31st January 2023. We compared their healthcare utilisation in the 12 months after index date (Fig. 1a). We included adults aged 18 or over who had been registered with a GP practice using TPP software for at least 3 months prior to 1st November 2020. To exclude bias due to unusual coding practices, we excluded patients registered with GP practices that did not use at least one long COVID diagnostic code between November 2020 and January 2023.

In the historical comparison, we took matched sets of patients from the contemporary comparison, who were additionally registered with their GP between March 2019 and the index date. Among the matched sets we examined (i) their historical healthcare utilisation between March 2019 and March 2020 and (ii) their contemporary utilisation after the index date (Fig. 1b). The difference in utilisation between exposed (long COVID) patients and unexposed patients was assessed using a difference-in-difference analysis.

### Exposures and comparators

The primary exposure of interest was long COVID, defined by SNOMED-CT codes recorded in primary care (including diagnostic, referral, and long COVID assessment codes [13] (Additional file 1: Table S1)). The date of the first long COVID code in the primary care records was defined as the index date. The exposures were matched to five non-long COVID comparators by age, sex, and region. Each comparator’s index date was assigned using their matched exposure's long COVID diagnostic date.

### Outcomes

For the contemporary comparison, the primary outcome was total healthcare resource utilisation in the 12 months following the index date. We followed the exposure and the matched comparators from the same index date to minimise the influence of different waves of the COVID-19 pandemic on healthcare utilisation [14]. Total healthcare resource utilisation was calculated by combining (1) primary care utilisation, including appointment with a GP and/or prescription of medications; (2) all-cause accident and emergency (A&E) visits, defined using A&E arrival records; (3) all-cause hospital admission, defined as admission to hospitals for more than 1 day; and (4) all-cause hospital outpatient clinic visits. For each type of healthcare utilisation, if there were multiple healthcare visits on the same date, such as receiving multiple prescriptions, they were counted as one visit. In addition, all appointments with GPs were considered the same, as detailed information, such as whether the consultation was over the phone, is not available.

The secondary outcome was the cumulative total healthcare costs in the 12 months after the index date. This was calculated by combining (1) primary care costs, including GP consultations and prescriptions; (2) hospitalisation costs; (3) A&E costs, and (4) outpatient clinic costs. We estimated the cost of a GP consultation by multiplying the GP visit counts and the average cost for a GP consultation in 2021/2022 (£41) [15]. To estimate the cost of GP prescriptions, we multiplied the frequency of prescriptions for each BNF chapter by the average cost of medications in that chapter in 2021/2022. The hospital admission costs, A&E costs, and hospital outpatient costs were provided by NHS England [16]. We additionally analysed the four components of utilisation and cost separately.

For the historical comparison, the outcome was the difference between total healthcare resource utilisations (as defined above) before (i.e. during the period March 2019–March 2020) and after being diagnosed with long COVID (12 months after index date).

### Covariates

In our analyses, we determined covariates by using a DAG (Additional file 2: Fig. S1), including age, sex, ethnicity, region and Index of Multiple Deprivation (IMD) quintile. We also included underlying chronic diseases, which included asthma, obesity/overweight, previous psychiatric conditions, and the level of multi-morbidity. The level of multi-morbidity was defined by categorising the number of chronic diseases listed in Additional file 3: Table S2. The covariate assessment period was 5 years before November 2020. We also considered previous hospital admissions due to COVID-19 and the number of COVID-19 vaccination doses (any vaccine) received before the index date.

### Statistical analysis

We first compared the distribution of demographic factors, underlying comorbidities, and socioeconomic factors in the long COVID and comparator groups. Categorical variables were assessed using Chi-square statistics, and the mean and standard deviation of continuous variables were compared using a t-test. In the contemporary comparison, because the distribution of healthcare visits and costs were zero-inflated and right-skewed (Additional file 4: Fig. S2a and 2b), we implemented a two-part model to analyse the healthcare utilisation and cost data [17, 18]. In brief, the first part of the model is a binomial model, estimating the probability of non-zero healthcare visits or non-zero healthcare costs; the second part of the model is a truncated GLM model conditioning on people with non-zero healthcare visits and non-zero healthcare costs. In the second part of the analysis, we used a negative binomial model to estimate the overall healthcare utilisation rate ratio and a Gamma GLM model to assess the total healthcare cost ratio. The observed follow-up time was included in both models as offsets. We examined the over-dispersion of the data by running a Poisson regression and examining the ratio between residual deviance and the degree of freedom. If the ratio was greater than 1, we applied a negative binomial model in the second part of our model and carried out a Poisson regression model if the ratio was close to 1. We further applied a prediction function to the regression model outputs, multiplying the probability of non-zero healthcare visits and the predicted healthcare visits, to obtain the predicted average healthcare utilisation and costs on the absolute scale.

For the historical comparison, we conducted a difference-in-difference (DID) analysis evaluating the change in healthcare utilisations before the pandemic compared to after a long COVID diagnosis. We created a time variable and categorised healthcare visits between March 2019 and March 2020 as “historical records” (pre-pandemic) and healthcare visits after the index date as “contemporary records”. By fitting this time variable interacting with the exposure variable in the two-part model, we could compare the healthcare utilisation difference before and after the index date within the exposure and the comparator groups, and then further calculate the difference between these two values. Similar to the contemporary comparison, we also used a prediction function to multiply the probability of non-zero healthcare visits and the predicted healthcare visits, to estimate the average healthcare visits before and after long COVID diagnoses on an absolute scale. The common trend assumption of DID was examined by comparing the average healthcare utilisation in the exposure and comparator groups over time (Additional file 5: Fig. S4).

### Sensitivity analysis

We carried out a series of sensitivity analyses. First, some patients in our study had healthcare visit records but the associated healthcare cost data were missing. We imputed missing secondary cost data by estimating the mean cost for one visit, appointment, or admission episode from people with both healthcare cost and healthcare visit records. Second, we stratified our analyses by sex, age group and previous hospital admission due to COVID-19. The stratum-specific results were obtained by fitting an interaction term between exposure and the stratifying variables. The interaction was examined using a likelihood ratio test. Third, because people with outcomes can only be identified if they visited a healthcare provider, we restricted the main analyses to people who had ever consulted a GP 1 year before the 1st of November 2020. Fourth, to balance the chance of getting long COVID between groups, we restricted the analyses to people who had tested positive for COVID. We also excluded the first GP visit record to accurately estimate the follow-up rates.

### Software and reproducibility

Data management was performed using Python 3.8, with analysis carried out using R 4.0. Code for data management and analysis as well as codelists archived online (https://github.com/opensafely/openprompt_health_utilisation). All iterations of the pre-specified study protocol are archived with version control (https://github.com/opensafely/openprompt_health_utilisation/blob/cd8ecce1e12018756375013cd3b27a25880d85a4/OpenPROMPT_longCOVID_healthcare_utilisation_protocol.pdf). We report our results following the RECORD reporting guideline [19] (Additional file 6: Table S3).

### Patient and Public Involvement and Engagement (PPIE)

In the OpenPROMPT research group, we have three representatives from the public through our Patient and Public Involvement and Engagement (PPIE) initiative. These representatives attended progress meetings every 6 months to provide feedback and insights on our work. Furthermore, we had two online open workshops inviting individuals living with long COVID, aiming to better understand their lived experiences and healthcare-seeking behaviours. In addition, OpenSAFELY has developed a publicly available website https://www.opensafely.org/, through which they invite any patient or member of the public to make contact regarding the broader OpenSAFELY project.

## Results

### Study population

The population selection process is summarised in Additional file 7: Fig. S5. We identified 52,988 people with long COVID and 264,867 matched comparators (Table 1). There were more females than males, and people aged 40 to 59 comprised 50% of both exposure and comparator groups. The long COVID group had a higher proportion of white ethnicity, obesity, asthma, mental health diseases, and other comorbidities, compared to the comparator group. The long COVID group were also more likely to have tested positive for COVID, have been hospitalised for COVID, and had received more COVID vaccines. Less than 40% of people in both groups had a linked positive SARS-CoV-2 test before the index date (Table 1). Compared with other covariates, there were more missing values for IMD quintile (1.8%), ethnicity (14.7%) and BMI categories (8.3%) (Additional file 8: Table S4). Approximately 2% of the participants who visited A&E had missing cost records, whereas the data for admission and A&E visit costs were relatively complete (Additional file 9: Table S5). The observed event counts and person-time by long COVID exposure were summarised in Additional file 10: Table S6.

**Table 1:** Distribution of demographic factors

Factors	Level	Total (*N*, %)	Long covid exposure (*N*, %)	Comparator (*N*, %)	*P*-value
Sex	Female	317,855 (100.0)	33,805 (63.8)	168,984 (63.8)	0.99
	Male		19,183 (36.2)	95,883 (36.2)	
Age	Mean (SD)	317,855 (100.0)	48.0 (14.4)	48.0 (14.4)	0.98
Age categories	18–29	317,855 (100.0)	6638 (12.5)	33,188 (12.5)	1.00
	30–39		10,050 (19.0)	50,235 (19.0)	
	40–49		13,241 (25.0)	66,196 (25.0)	
	50–59		13,236 (25.0)	66,153 (25.0)	
	60–69		6199 (11.7)	30,984 (11.7)	
	70 +		3624 (6.8)	18,111 (6.8)	
Ethnicity	White	271,230 (85.3)	40,972 (89.0)	195,292 (86.7)	< 0.01
	Mixed		539 (1.2)	2801 (1.2)	
	South Asian		3053 (6.6)	16,227 (7.2)	
	Black		832 (1.8)	5622 (2.5)	
	Other		655 (1.4)	5237 (2.3)	
BMI categories	Normal weight	291,349 (91.7)	854 (1.7)	5334 (2.2)	< 0.001
	Underweight		13,310 (26.4)	79,909 (33.2)	
	Overweight		15,802 (31.4)	78,873 (32.7)	
	Obese		20,436 (40.5)	76,831 (31.9)	
Index of multiple deprivation (IMD)	least deprived	312,032 (98.2)	10,457 (20.1)	52,359 (20.1)	0.93
	2nd deprived		10,446 (20.1)	52,341 (20.1)	
	3rd deprived		10,478 (20.1)	51,978 (20.0)	
	4th deprived		10,349 (19.9)	52,021 (20.0)	
	Most deprived		10,272 (19.8)	51,331 (19.7)	
Region	East	317,840 (100.0)	10,162 (19.2)	50,807 (19.2)	1.00
	East Midlands		7513 (14.2)	37,562 (14.2)	
	London		2400 (4.5)	11,995 (4.5)	
	North East		4161 (7.9)	20,805 (7.9)	
	North West		6068 (11.5)	30,340 (11.5)	
	South East		3707 (7.0)	18,535 (7.0)	
	South West		8267 (15.6)	41,353 (15.6)	
	West Midlands		1675 (3.2)	8374 (3.2)	
	Yorkshire and The Humber		9020 (17.0)	45,096 (17.0)	
Asthma	No asthma	317,855 (100.0)	40,618 (76.7)	223,151 (84.3)	< 0.01
	Have asthma		12,370 (23.3)	41,716 (15.7)	
Mental health issues	No mental health issues	317,855 (100.0)	34,320 (64.8)	201,189 (76.0)	< 0.01
	Have mental health issues		18,668 (35.2)	63,678 (24.0)	
Number of comorbidities	0	317,855 (100.0)	43,476 (82.0)	225,058 (85.0)	< 0.01
	1		7997 (15.1)	34,450 (13.0)	
	2		1296 (2.4)	4,737 (1.8)	
	3 or more		219 (0.4)	622 (0.2)	
Previous hospitalisation due to COVID	No	317,855 (100.0)	48,333 (91.2)	263,162 (99.4)	< 0.01
	Yes		4655 (8.8)	1705 (0.6)	
Number of COVID vaccine received at index date	0 dose	317,855 (100.0)	8454 (16.0)	54,698 (20.7)	< 0.01
	1 dose		5215 (9.8)	23,771 (9.0)	
	2 doses		17,259 (32.6)	80,899 (30.5)	
	3 or more doses		22,060 (41.6)	105,499 (39.8)	
Had long COVID diagnoses	No	317,855 (100.0)	0 (0.0)	264,270 (99.8)	< 0.01
	Yes		52,988 (100.0)	597 (0.2)	
Had been tested positive for COVID-19	No	317,855 (100.0)	32,006 (60.4)	182,448 (68.9)	< 0.01
	Yes		20,982 (39.6)	82,419 (31.1)	

### Healthcare utilisation and cost among people with long COVID

**Fig. 2:**
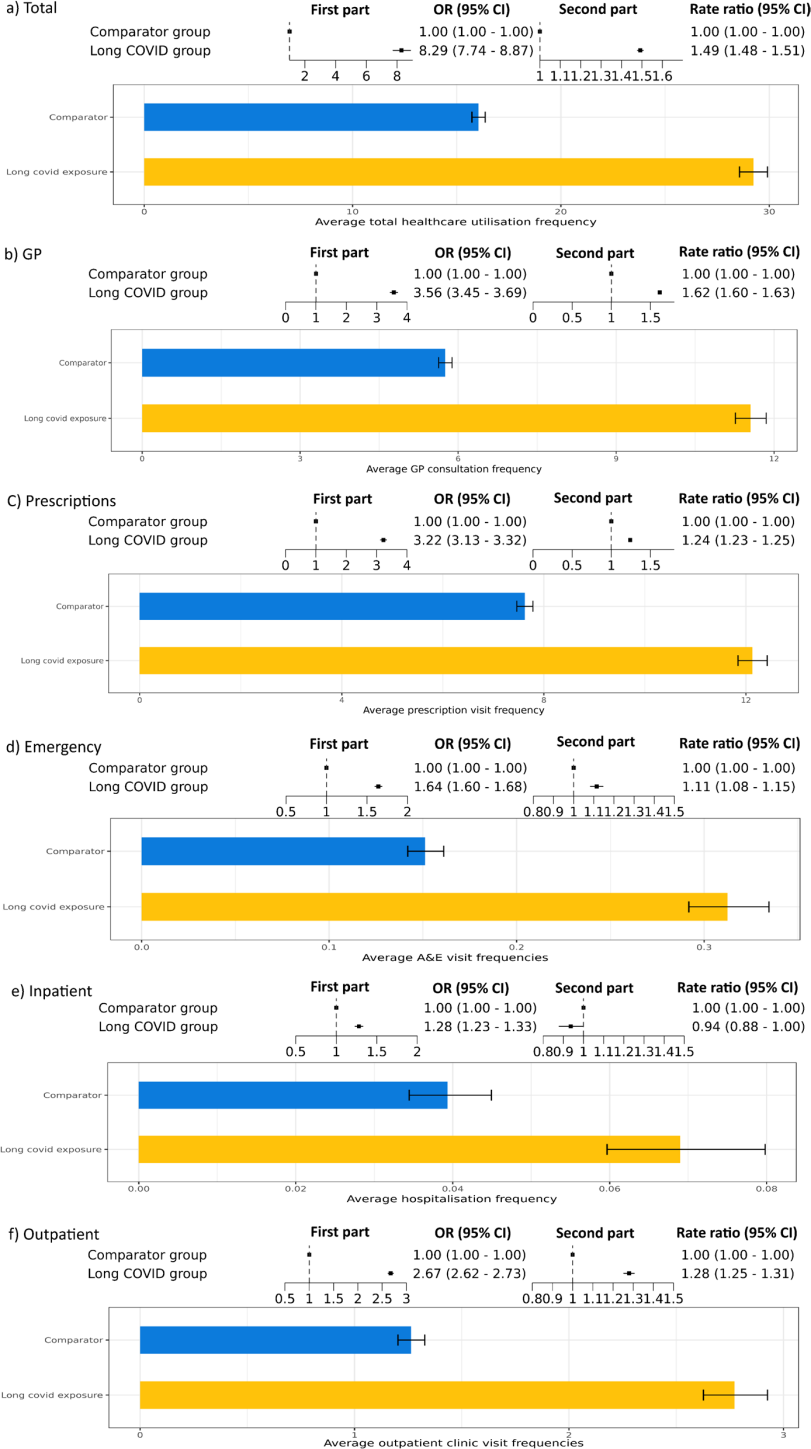
Healthcare utilisation among people with long COVID. Each subfigure shows the first and second part of the model in the upper panel, where the first part gives the odds ratio (OR) of healthcare resource utilisation and the second part is the rate ratio (RR) of healthcare utilisation between people with long COVID and comparator groups, conditioned on people having used healthcare resources. Each lower panel shows the predicted average healthcare utilisation in long COVID and Comparator groups. Values are shown for **a** total healthcare utilisation, and then separately for each part of the total: **b** GP visits, **c** prescriptions, **d** emergency care at A&E, **e** inpatient hospitalisations, and **f** outpatient visits

After adjusting for covariates, there was strong evidence that people with long COVID were more likely to use healthcare resources (Fig. 2a, first part: odds ratio (OR): 8.29, 95% CI: 7.74–8.87), and further among those who visited their healthcare providers, there was strong evidence that people with long COVID had a higher rate of total healthcare utilisation (Fig. 2a, second part: rate ratio (RR): 1.49, 95% CI: 1.48–1.51). The predicted model shows that on average, the long COVID group had nearly 30 healthcare visits per year (predicted mean: 29.23, 95% CI: 28.58–29.92), while the comparator group had 16 visits per year (predicted mean visits: 16.04, 95% CI: 15.73–16.36) (Fig. 2, average total healthcare utilisations). The increase in healthcare utilisation persisted across most healthcare types (Fig. 2b–d,f). For inpatient stays, people with long COVID were more likely to be hospitalised (OR 1.28, 95% CI: 1.23–1.33), while there was some evidence that the hospitalisation rate ratio was slightly lower (RR: 0.94, 95% CI: 0.88–1.00). However, the predicted hospitalisation counts of the long COVID group were still larger than the comparator group (Fig. 2e).

**Fig. 3:**
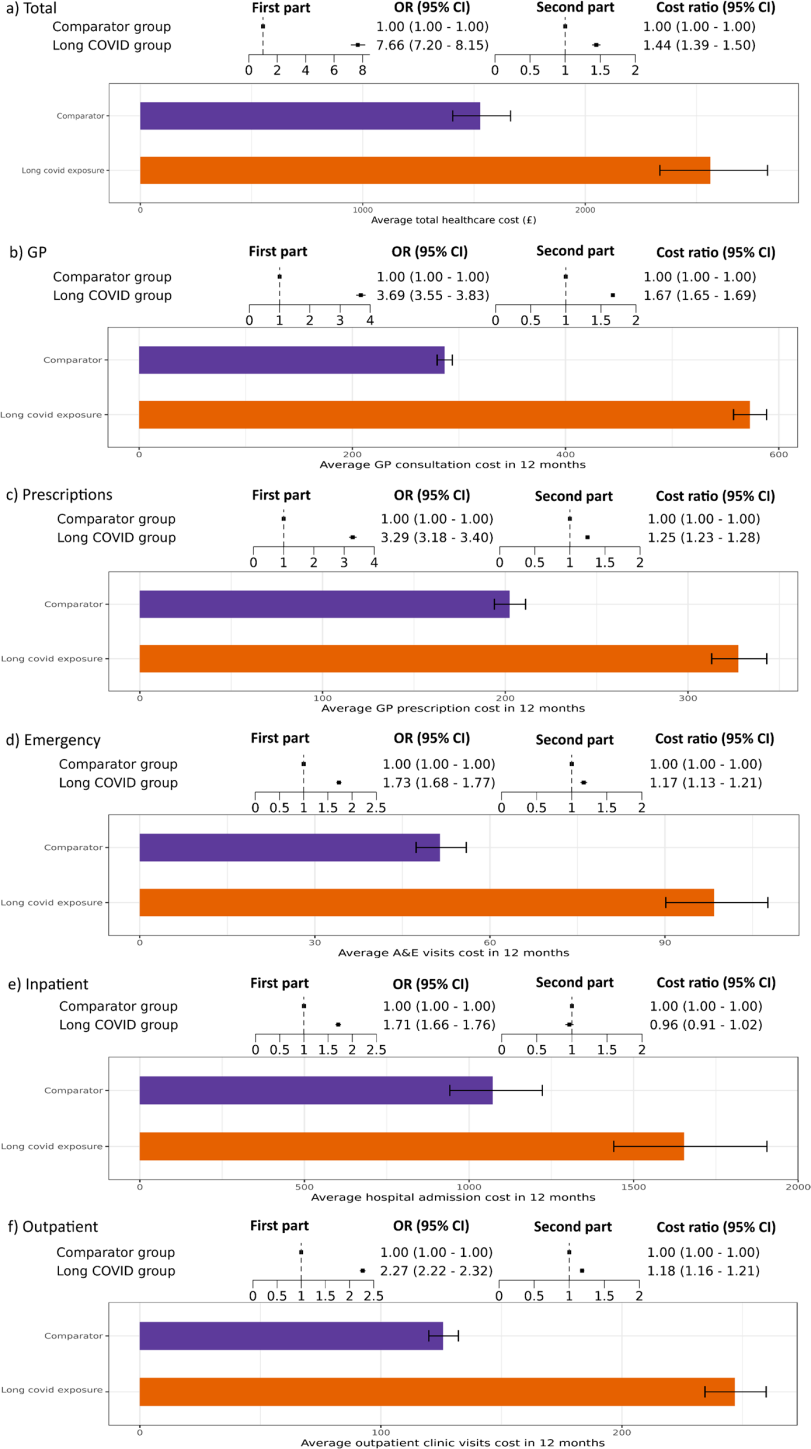
Total health costs among people with and without long COVID. Each subfigure shows the first and second part of the model in the upper panel, where the first part is the odds ratio (OR) of having any healthcare costs and the second part is the rate ratio (RR) of healthcare costs between people with long COVID and comparator groups, conditioned on people having any healthcare costs. Each bar chart shows the predicted average healthcare costs among the long COVID and Comparator groups. Values are shown for **a** total healthcare costs, and then separately for each part of the total: **b** GP visits, **c** prescriptions, **d** emergency care at A&E, **e** inpatient hospitalisations, and **f** outpatient visits

We also estimated healthcare costs; after adjusting for covariates, there was strong evidence that people with long COVID were seven times more likely to have non-zero healthcare costs (Fig. 3a, first part, OR = 7.66, 95% CI = 7.20–8.15). Among people with non-zero healthcare costs, the total costs for people with long COVID were 44% higher than those of the comparator groups (Fig. 3a, second part, cost ratio = 1.44, 95% CI: 1.39–1.50). The predicted model showed that costs for the long COVID group were approximately £2500 per person per year (predicted mean cost: £2562.50, 95% CI: £2335.60–£2819.22), and £1500 in the comparator group (predicted mean cost: £1527.43, 95% CI: £1404.33–1,664.45) (Fig. 3a, average total healthcare cost). The increase in healthcare costs persisted across all healthcare types (Fig. 3b–f) and as for healthcare utilisation, in those using any healthcare services, the cost ratio was highest for GPs and lowest for inpatient hospital stays.

### Historical comparison

**Fig. 4:**
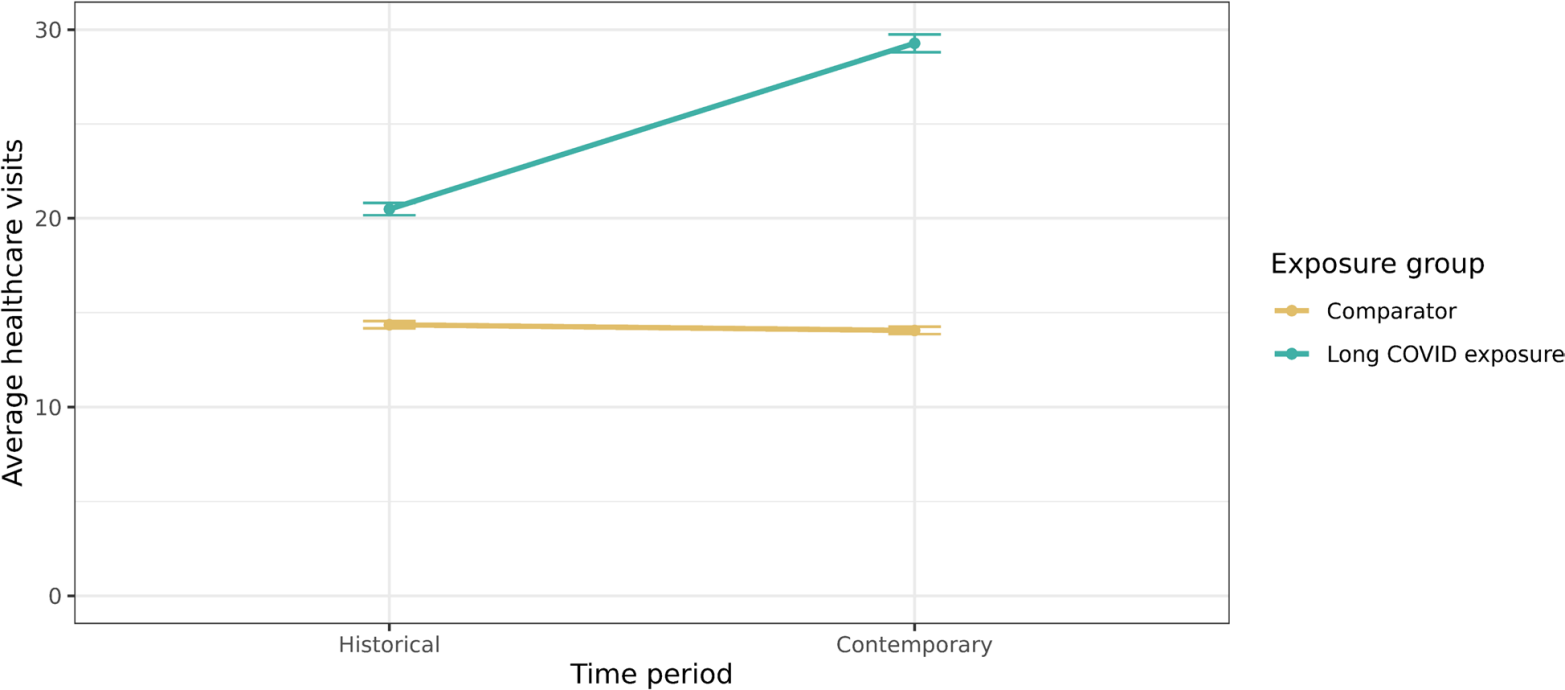
Predicted average healthcare visits before and after the pandemic. The analysis is based on a difference-in-difference analysis comparing those with long COVID and their age-, sex- and region-matched comparators

The difference-in-difference analyses demonstrated that individuals with a long COVID diagnosis had historically higher healthcare utilisation compared to controls, but that this difference became more marked after a long COVID diagnosis. Before the pandemic, there were approximately 20 predicted healthcare visits among the group who went on to be diagnosed with long COVID (predicted mean visits: 20.48, 95% CI:20.16–20.81), and 14 in the comparator group (predicted mean visits: 14.35, 95% CI:14.15–15.55). After the pandemic, total healthcare utilisation increased in the long COVID group to 29 (predicted mean visits: 29.28, 95% CI: 28.81–29.75), but remained at 14 in the comparator group (predicted mean visits: 14.05, 95% CI:13.85–14.24) (Fig. 4).

### Factors associated with high healthcare use

**Fig. 5:**
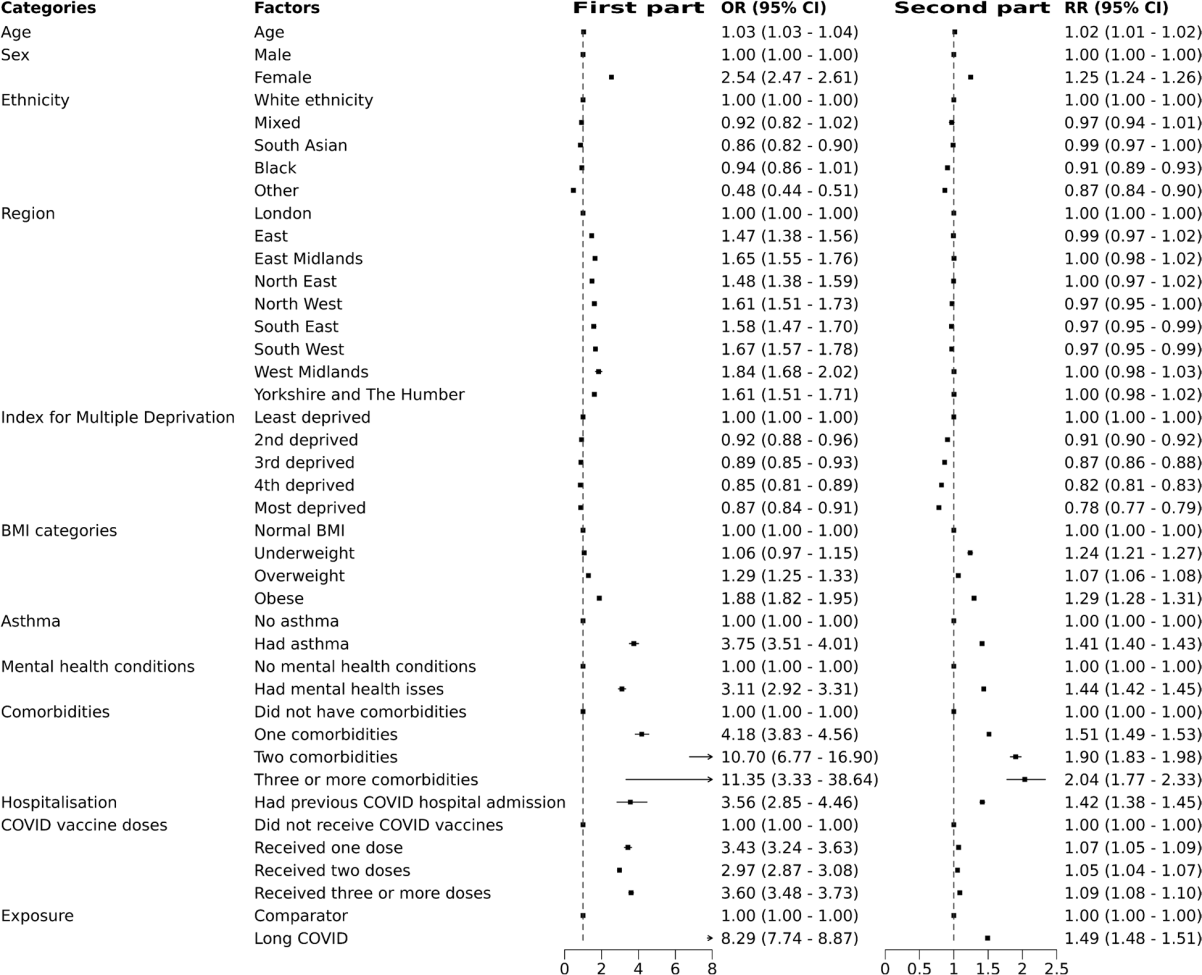
Factors associated with high healthcare use from the two-part model. The first forest plot shows the odds ratio (OR) of having non-zero healthcare use from the binomial model, the second part is the rate ratio (RR) for healthcare use from the truncated negative binomial model

In the fully-adjusted analysis model considering long COVID, we found that female sex, being obese, having asthma or mental health issues, having more comorbidities, and being previously admitted to hospital due to COVID were consistently associated with increased healthcare utilisation in both parts of the model (Fig. 5).

### Sensitivity analysis

After imputing missing cost data, the odds of non-zero healthcare costs and the cost ratio increased for long COVID patients, despite a slight decrease in average total cost (Additional file 11: Fig. S5). In our stratified analyses, the long COVID exposure group had a higher rate ratio of non-zero healthcare visits, which was more pronounced among individuals who had been hospitalised due to COVID, female sex, and those aged 30 to 69 (Additional file 12: Fig. S6). However, when conditioned on non-zero healthcare resource utilisation, although the long COVID exposure group still had a higher healthcare utilisation rate than the comparator group, the rate ratio was lower among the previously hospitalised and people aged over 70 strata (Additional file 12: Fig. S6a). Females and people aged 18 to 69 had a higher rate ratio (Additional file 12: Fig. S6b and 6c). The predicted average healthcare visits were still higher among previously admitted individuals, female sex, and older adults (Additional file 12: Fig. S6). After restricting our analyses to individuals who had been registered to a GP for 1 year, those who had previously tested positive, or excluding the first GP records, we continued to observe increased healthcare utilisations among individuals with long COVID, compared to our matched comparator group (Additional file 13 Fig. S7, Additional file 14 Fig. S8, and Additional file 15 Fig. S9).

## Discussion

Our study revealed an increase in overall healthcare utilisation and associated costs in the year following a long COVID diagnosis, in comparison to those without recorded long COVID. This increase was observed across primary and secondary care including A&E visits, outpatient and inpatient stays. Our fully-adjusted models predicted that those with long COVID had nearly 30 healthcare visits per year, while the comparator group had 16 visits per year. The majority of visits in both groups were for attendance in primary care and receipt of prescriptions. The associated cost to the NHS was found to be approximately £2500 in the long COVID group, and £1500 in the comparators.

Our historical comparison demonstrated that those with long COVID were more likely to be higher users of healthcare before the pandemic compared to comparators. This indicates that those with long COVID likely had a higher pre-existing comorbidity burden than their matched comparators, as demonstrated by the differences between groups in Table 1. The change in utilisation among those diagnosed with long COVID was far greater than the change in the comparator group, indicating that long COVID may have been responsible for the increase in consultation and costs that we observed, and also highlighting the importance of adjusting for comorbidity burden in the contemporary comparison. Finally, we found that factors associated with healthcare utilisation included female sex, a history of asthma or mental health conditions, presence of comorbidities, and prior hospitalisation due to COVID-19.

Our findings relating to utilisation and costs are consistent with studies in other healthcare settings for healthcare utilisation after a COVID-19 diagnosis [4–6], and for associated healthcare costs [9]. In the US, a study using Medicare data reported that among people aged over 65, people with long COVID had a higher risk of hospitalisations and outpatient visits for any cause, compared with the historical comparator group with long-term influenza symptoms [20]. A study from Israel found individuals with long COVID had a higher risk of hospitalisation, home hospitalisation, and emergency visits, and an increase in costs [8], mirroring similar pre-printed findings in England [21].

A possible explanation for increased utilisation and cost is that people with long COVID attend healthcare settings separately for a variety of symptoms, for example affecting the respiratory, cardiovascular, and central nervous systems, and general non-specific symptoms. These are more likely to be reported in primary care [6, 22], which could contribute to our finding of the highest primary care resource use. While effect sizes for secondary care were similar to primary care, the relatively low utilisation across A&E, outpatient and inpatient visits may relate to the nature of long COVID symptoms and the lack of effective treatment. GPs are able to offer referrals to long COVID clinics, where these are available, but any further effective treatments have so far not been identified. In addition, we also acknowledge that media coverage can influence individuals’ healthcare-seeking behaviours. For instance, a previous study on Group A Streptococcal (GAS) diseases found a correlation between extensive media coverage and increased rates of GAS testing [23]. Therefore, raising awareness about long COVID may also contribute to an increase in healthcare utilisation among people with long COVID.

Our findings on factors associated with high utilisation support a recent systematic review which reported that female sex, older age, and hospitalisation, including ICU admission, are risk factors for developing post-COVID conditions [22], and are associated with high healthcare use more generally [24]. In addition, in our sensitivity analyses, previous COVID-related hospitalisation, sex, and age group further modified the association between long COVID and healthcare utilisation.

Our study uses a large, representative EHR sample of individuals with clinically-recorded long COVID, and we used statistical methods for estimating healthcare utilisation and costs appropriate for zero-inflated data [17]. Advancing previous analyses, we followed people with a long COVID diagnosis, and for a year post-diagnosis to ascertain the full impact of long COVID on healthcare utilisation. We did not restrict to individuals with a positive COVID-19 test (similar to other studies) because we found that only a fraction of people with long COVID diagnoses had previously tested positive in previous work [25]. However, in sensitivity analyses, we found that the results remained similar.

Key limitations are that the exposure and the outcomes were identified from EHR databases, which depend on people being registered and visiting their healthcare service providers. To address this, we conducted a sensitivity analysis among people who registered at least 1 year before the study follow-up and had at least one GP consultation record, and the results remained similar. Further, people with long COVID who do not have an EHR code could be misclassified to the comparator group. In addition, we were unable to disentangle the effect of increased morbidity due to long COVID from possible undiagnosed conditions prior to COVID. A limitation of our economic analysis is that we did not have directly collected primary care data, and some cost data for secondary care were also missing. For primary care, we used the reported average cost for a GP consultation, as in other health economic studies [26]. For secondary care, in a sensitivity analysis, we imputed the missing data and the results remained similar.

We aimed here to examine NHS costs, but our patient advisory group suggested that people with long COVID frequently seek private healthcare, if they are able, and therefore, our estimates of utilisation and cost will be underestimated, and importantly the use of private healthcare might exacerbate any socioeconomic inequalities in care. The extent of private healthcare use and wider societal costs of long COVID could not be captured in this study.

## Conclusions

Our study found that people with long COVID had increased healthcare utilisation and costs across all healthcare sectors, compared with people without long COVID. Differences varied by type of healthcare utilisation but persisted across all sensitivity analyses. We showed that people with a long COVID diagnosis typically had a higher historical healthcare burden, but that a long COVID diagnosis greatly increased their utilisation and the associated cost to the health service. Our study contributes to the growing body of evidence demonstrating the impact of long COVID, in terms of quality of life, use of healthcare, and cost. Long COVID may also affect people’s ability to participate in the workforce, with further economic consequences as well as inducing direct costs to affected individuals. Our results have implications for resource planning in future waves of infection. For example, when planning future vaccination programmes and policies for mitigating the effects of COVID, the impact of long COVID on the NHS and wider economy should be considered in addition to that of the acute COVID illness itself. Our findings imply that long COVID poses a considerable burden on attendance at all healthcare facilities and induces major healthcare costs for affected patients. Public health policies need to allocate resources for the prevention, treatment, and support of people with long COVID.

### Supplementary Information

Additional file 1.

Additional file 2.

Additional file 3.

Additional file 4.

Additional file 5.

Additional file 6.

Additional file 7.

Additional file 8.

Additional file 9.

Additional file 10.

Additional file 11.

Additional file 12.

Additional file 13.

Additional file 14.

Additional file 15.

## Data Availability

NHS England is the data controller of the NHS England OpenSAFELY COVID-19 Service; TPP is the data processor; all study authors using OpenSAFELY have the approval of NHS England [27]. This implementation of OpenSAFELY is hosted within the TPP environment which is accredited to the ISO 27001 information security standard and is NHS IG Toolkit compliant [28]. Other researchers can apply to access OpenSAFELY (see OpenSAFELY.org). We uploaded our analysis codes to GitHub: https://github.com/opensafely/openprompt_health_utilisation.

## Abbreviations

A&E: Acute and emergency care
EHRs: Electronic health records
GP: General practice
NHS: National Health Service
OR: Odds ratio
RR: Rate ratio
TPP: The Phoenix Partnership
