# Supplementary material for: Healthcare utilisation in people with long COVID: an OpenSAFELY cohort study
Source: BMC Med. 2024 Jun 20;22:255. doi: 10.1186/s12916-024-03477-x (PMC11188519; doi:10.1186/s12916-024-03477-x)
Supplement: Supplementary file 1 — Additional file 1. [file 12916_2024_3477_MOESM1_ESM.docx]

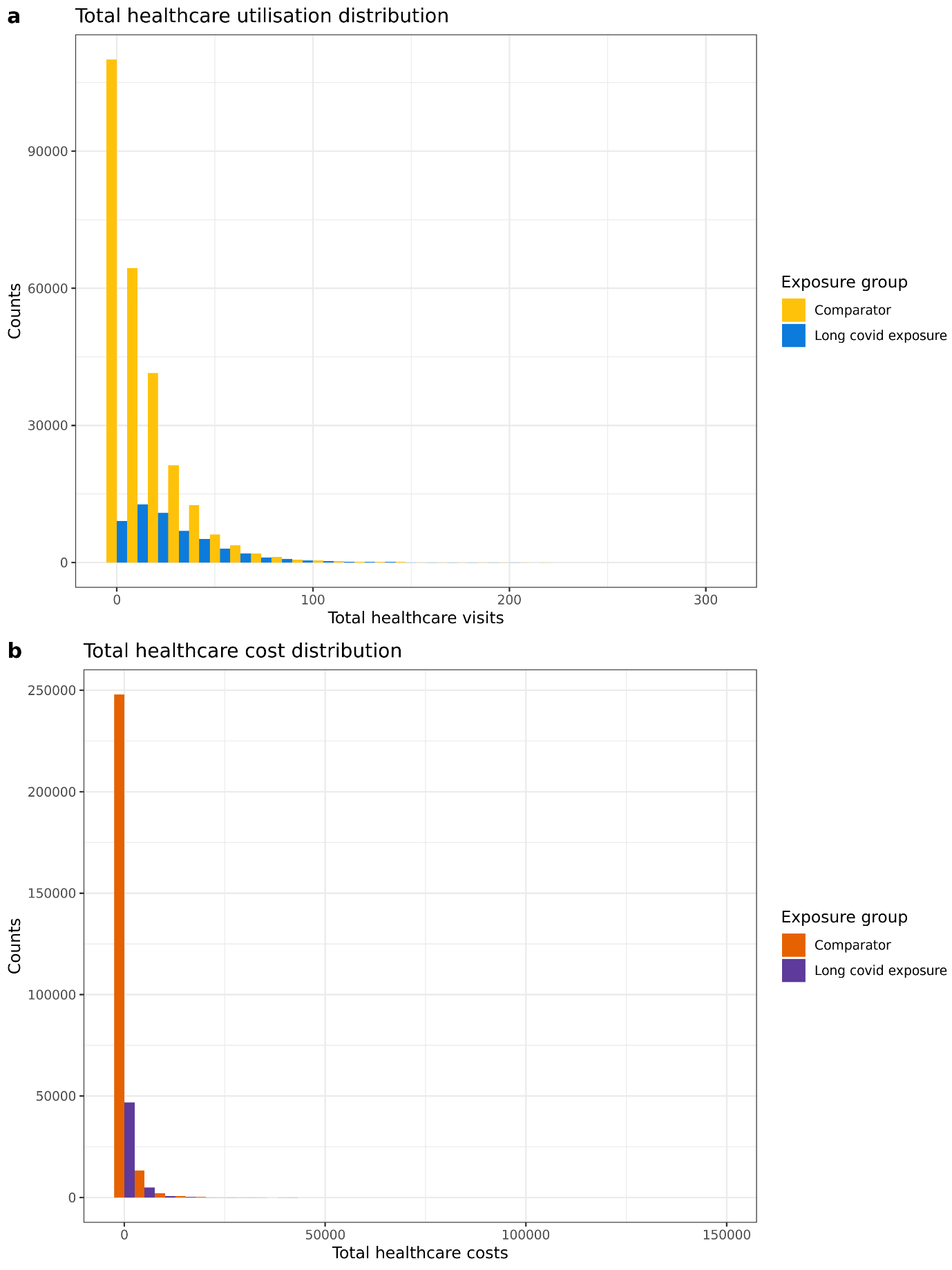


### Fig. S2. Distribution of healthcare utilisation and costs by exposure group. a. the distribution of healthcare visits; b. the distribution of healthcare costs.
