# Supplementary material for: Healthcare utilisation in people with long COVID: an OpenSAFELY cohort study
Source: BMC Med. 2024 Jun 20;22:255. doi: 10.1186/s12916-024-03477-x (PMC11188519; doi:10.1186/s12916-024-03477-x)
Supplement: Supplementary file 2 — Additional file 2. [file 12916_2024_3477_MOESM2_ESM.docx]

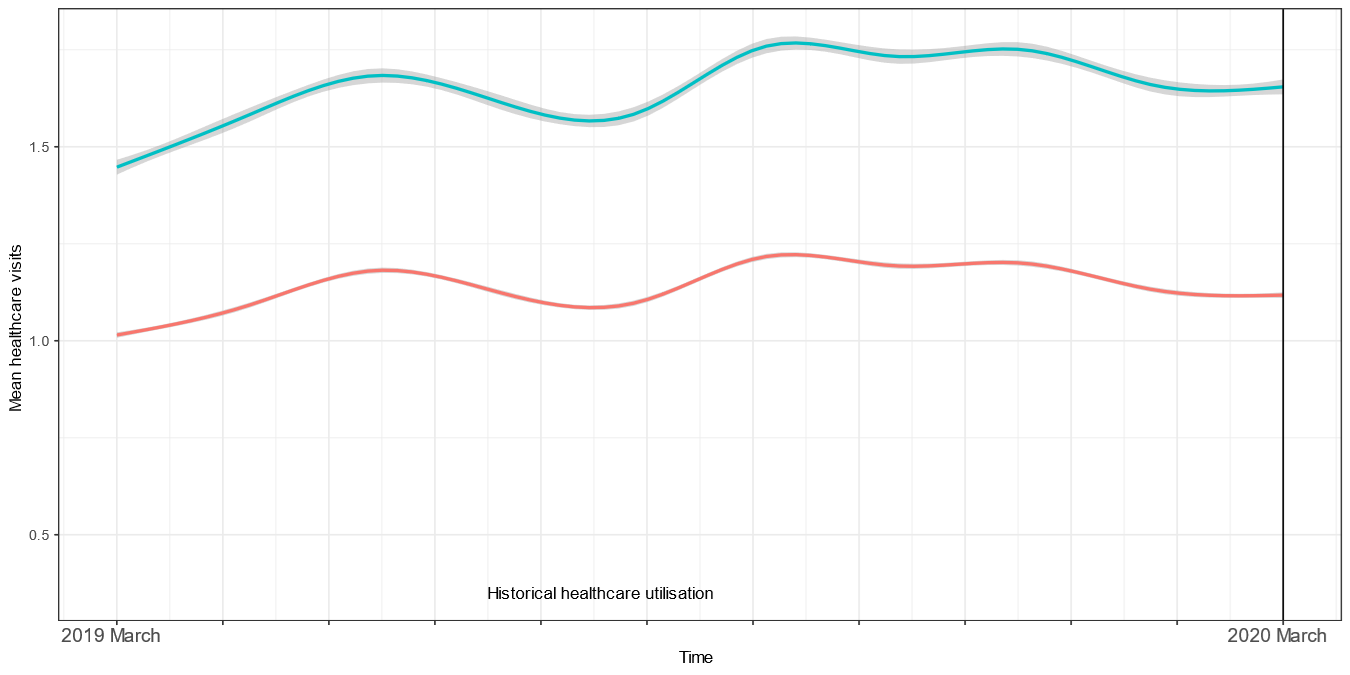


### Fig. S4. Historical average healthcare visits. The trend in healthcare utilisation for the long COVID and comparator groups was parallel before the pandemic, meeting the common trend assumption.
