# Supplementary figures and images for: Healthcare utilisation in people with long COVID: an OpenSAFELY cohort study
Source: BMC Med. 2024 Jun 20;22:255. doi: 10.1186/s12916-024-03477-x (PMC11188519; doi:10.1186/s12916-024-03477-x)

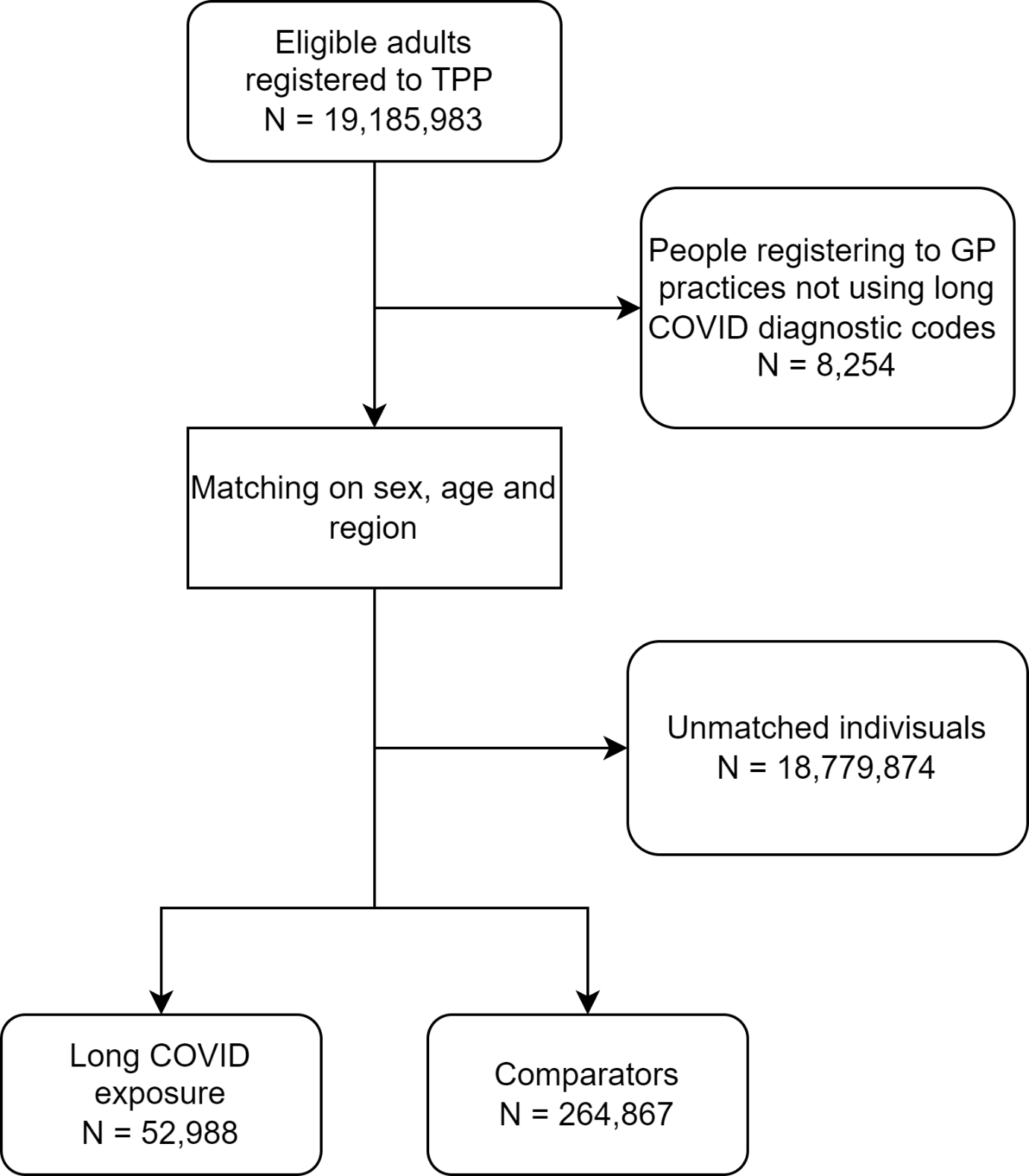


### Fig S5. The flowchart of selecting the study population

Supplement: Supplementary file 4 — Additional file 4. [file 12916_2024_3477_MOESM4_ESM.docx]

###
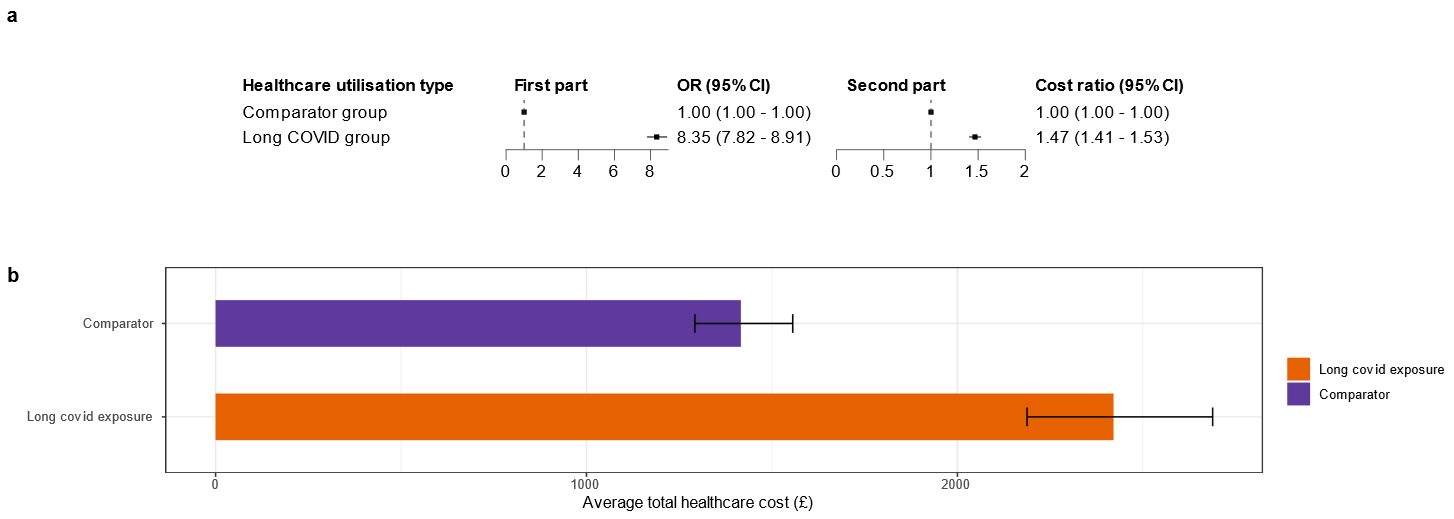
 Fig. S5. Imputed total healthcare cost

Supplement: Supplementary file 8 — Additional file 8. [file 12916_2024_3477_MOESM8_ESM.docx]

###
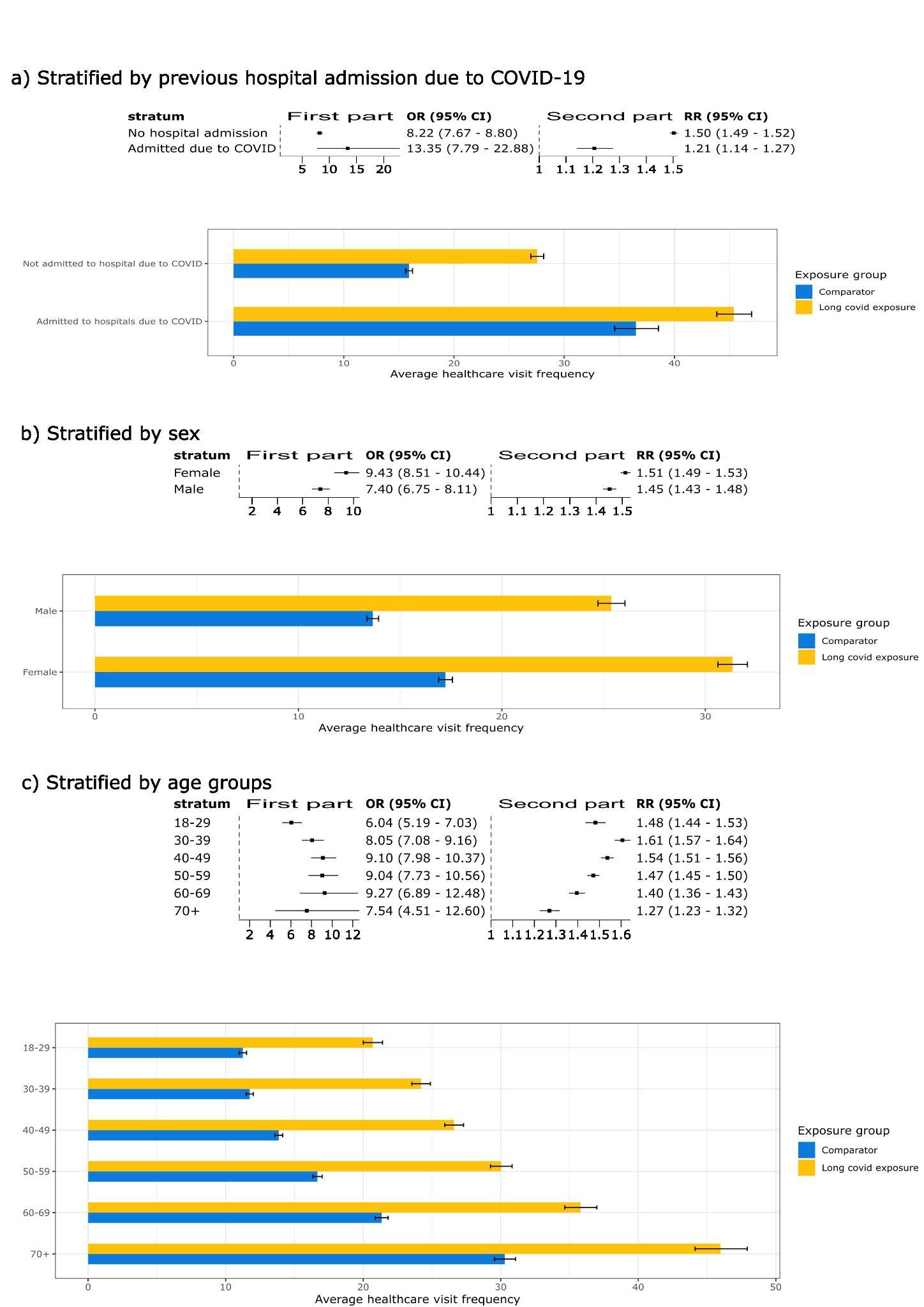
 Fig. S6. Stratified analysis by variables

Supplement: Supplementary file 9 — Additional file 9. [file 12916_2024_3477_MOESM9_ESM.docx]

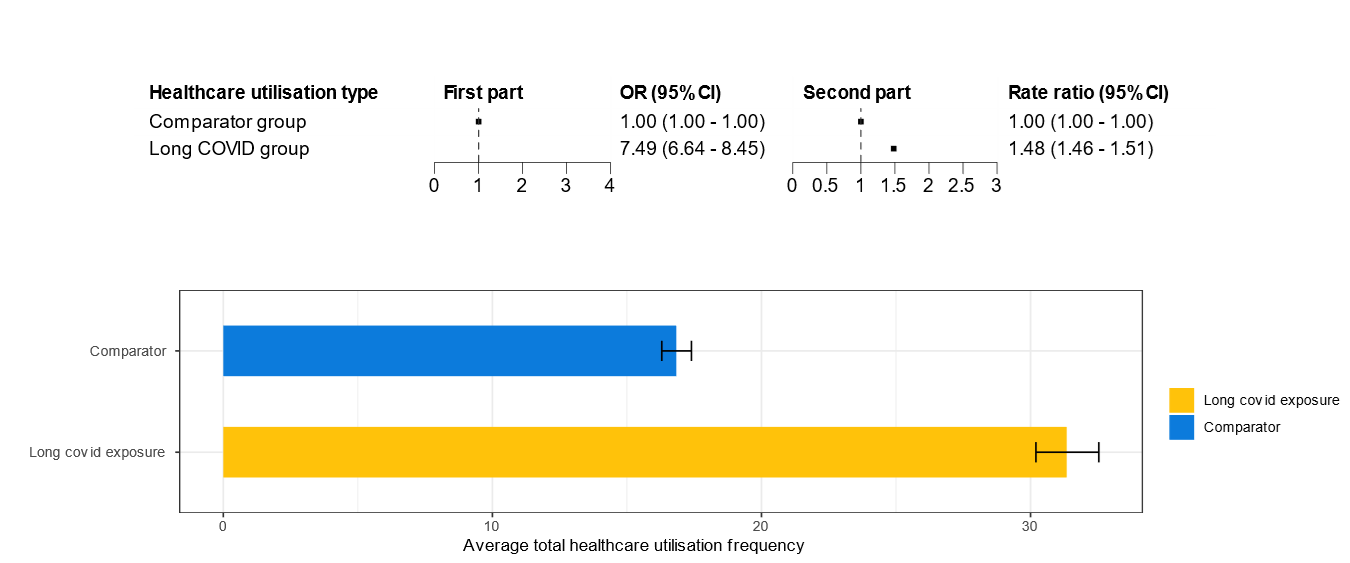


**Fig. S8**. Analyses among people who had tested positive for COVID before the index date

Supplement: Supplementary file 11 — Additional file 11. [file 12916_2024_3477_MOESM11_ESM.docx]

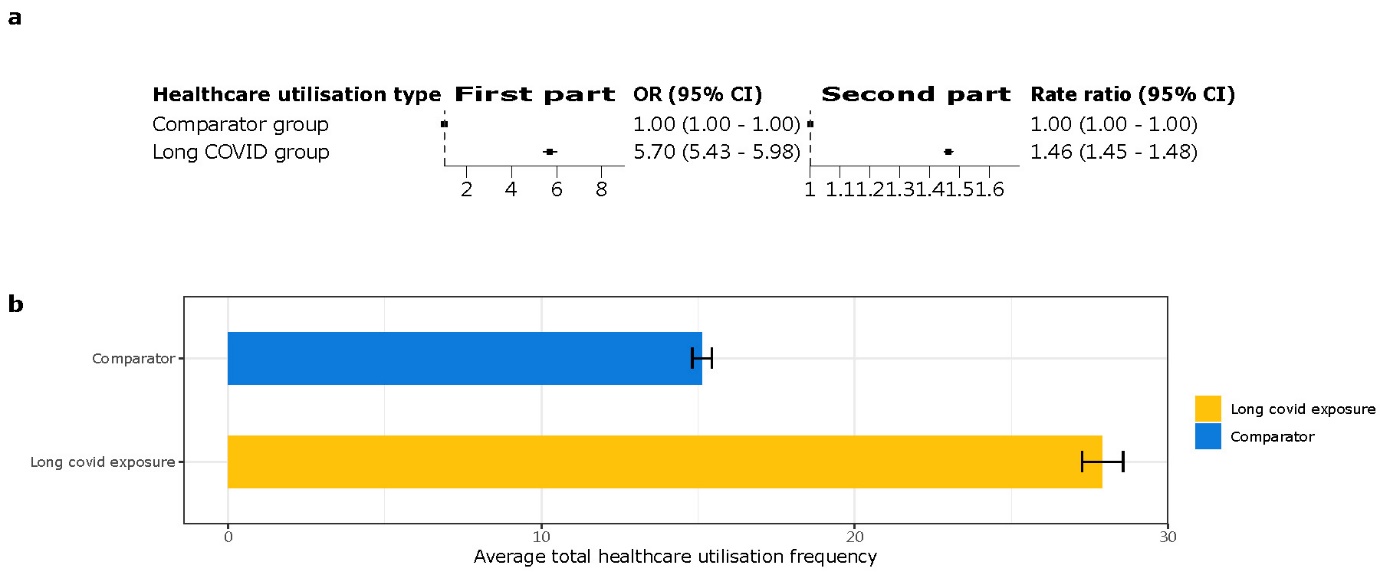


**Fig. S9.** Sensitivity analyses excluding the first GP appointment record

Supplement: Supplementary file 12 — Additional file 12. [file 12916_2024_3477_MOESM12_ESM.docx]

### Fig. S1. DAGs for covariate selection.
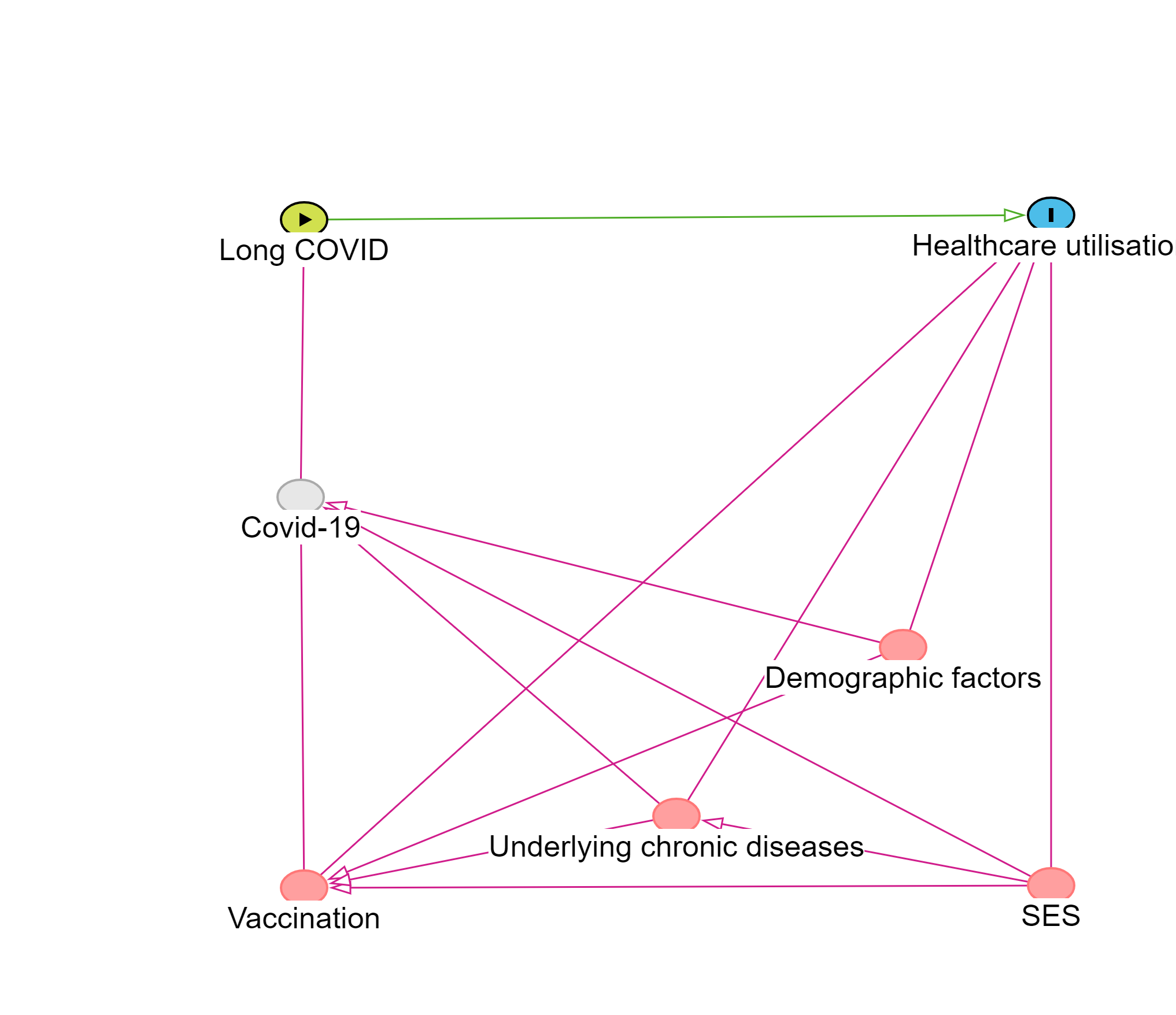

Supplement: Supplementary file 14 — Additional file 14. [file 12916_2024_3477_MOESM14_ESM.docx]
