# Supplementary material for: Healthcare utilisation in people with long COVID: an OpenSAFELY cohort study
Source: BMC Med. 2024 Jun 20;22:255. doi: 10.1186/s12916-024-03477-x (PMC11188519; doi:10.1186/s12916-024-03477-x)
Supplement: Supplementary file 5 — Additional file 5. [file 12916_2024_3477_MOESM5_ESM.docx]

### Table S4. Missing value across variables.

| **Variable** | **Total number** | **Missing number** | **Missing percentage (%)** |
| --- | --- | --- | --- |
| **Age** | 317,852 | 0 | 0 |
| **Sex** | 317,852 | 0 | 0 |
| **Region** | 317,837 | 15 | 0 |
| **IMD quintile** | 312,029 | 5,823 | 1.8 |
| **Ethnicity categories** | 271,276 | 46,576 | 14.7 |
| **BMI categories** | 291,410 | 26,442 | 8.3 |
| **Long COVID diagnoses** | 317,852 | 0 | 0 |
| **COVID test positive** | 317,852 | 0 | 0 |
| **Previous COVID-19 hospitalisation** | 317,852 | 0 | 0 |
| **Mental health issues** | 317,852 | 0 | 0 |
| **Asthma** | 317,852 | 0 | 0 |
| **Number of comorbidities** | 317,852 | 0 | 0 |
| **Number of COVID vaccine received** | 317,852 | 0 | 0 |
