# Supplementary material for: Healthcare utilisation in people with long COVID: an OpenSAFELY cohort study
Source: BMC Med. 2024 Jun 20;22:255. doi: 10.1186/s12916-024-03477-x (PMC11188519; doi:10.1186/s12916-024-03477-x)
Supplement: Supplementary file 6 — Additional file 6. [file 12916_2024_3477_MOESM6_ESM.docx]

### Table S5. Distribution of outcome variables.

| **Outcome type** | **Month** | **Total number** | **Missing number** | **Long covid exposure** | **Comparator** | **p-value** |
| --- | --- | --- | --- | --- | --- | --- |
| **Mean total healthcare visits** | 1 | 317,852 (100.0) | 0 | 3.2 (2.9) | 1.3 (2.0) | <0.001 |
|  | 2 | 317,852 (100.0) | 0 | 2.3 (2.6) | 1.2 (1.9) | <0.001 |
|  | 3 | 317,852 (100.0) | 0 | 2.2 (2.5) | 1.2 (1.9) | <0.001 |
|  | 4 | 317,852 (100.0) | 0 | 2.1 (2.5) | 1.2 (1.9) | <0.001 |
|  | 5 | 317,852 (100.0) | 0 | 2.0 (2.5) | 1.1 (1.9) | <0.001 |
|  | 6 | 317,852 (100.0) | 0 | 1.9 (2.4) | 1.1 (1.8) | <0.001 |
|  | 7 | 317,852 (100.0) | 0 | 1.8 (2.4) | 1.1 (1.8) | <0.001 |
|  | 8 | 317,852 (100.0) | 0 | 1.8 (2.5) | 1.0 (1.8) | <0.001 |
|  | 9 | 317,852 (100.0) | 0 | 1.6 (2.3) | 1.0 (1.8) | <0.001 |
|  | 10 | 317,852 (100.0) | 0 | 1.5 (2.3) | 0.9 (1.7) | <0.001 |
|  | 11 | 317,852 (100.0) | 0 | 1.5 (2.3) | 0.9 (1.7) | <0.001 |
|  | 12 | 317,852 (100.0) | 0 | 1.3 (2.1) | 0.8 (1.6) | <0.001 |
| **Mean GP consultations** | 1 | 317,852 (100.0) | 0 | 1.7 (1.7) | 0.5 (1.0) | <0.001 |
|  | 2 | 317,852 (100.0) | 0 | 1.0 (1.4) | 0.5 (1.0) | <0.001 |
|  | 3 | 317,852 (100.0) | 0 | 0.9 (1.3) | 0.5 (1.0) | <0.001 |
|  | 4 | 317,852 (100.0) | 0 | 0.8 (1.3) | 0.5 (1.0) | <0.001 |
|  | 5 | 317,852 (100.0) | 0 | 0.8 (1.3) | 0.4 (1.0) | <0.001 |
|  | 6 | 317,852 (100.0) | 0 | 0.7 (1.2) | 0.4 (0.9) | <0.001 |
|  | 7 | 317,852 (100.0) | 0 | 0.7 (1.2) | 0.4 (0.9) | <0.001 |
|  | 8 | 317,852 (100.0) | 0 | 0.7 (1.2) | 0.4 (0.9) | <0.001 |
|  | 9 | 317,852 (100.0) | 0 | 0.6 (1.2) | 0.4 (0.9) | <0.001 |
|  | 10 | 317,852 (100.0) | 0 | 0.6 (1.2) | 0.4 (0.9) | <0.001 |
|  | 11 | 317,852 (100.0) | 0 | 0.6 (1.1) | 0.3 (0.9) | <0.001 |
|  | 12 | 317,852 (100.0) | 0 | 0.5 (1.1) | 0.3 (0.8) | <0.001 |
| **Mean prescription visits** | 1 | 317,852 (100.0) | 0 | 1.1 (1.3) | 0.6 (0.9) | <0.001 |
|  | 2 | 317,852 (100.0) | 0 | 1.0 (1.2) | 0.6 (0.9) | <0.001 |
|  | 3 | 317,852 (100.0) | 0 | 0.9 (1.2) | 0.6 (0.9) | <0.001 |
|  | 4 | 317,852 (100.0) | 0 | 0.9 (1.2) | 0.6 (0.9) | <0.001 |
|  | 5 | 317,852 (100.0) | 0 | 0.9 (1.2) | 0.5 (0.9) | <0.001 |
|  | 6 | 317,852 (100.0) | 0 | 0.8 (1.1) | 0.5 (0.9) | <0.001 |
|  | 7 | 317,852 (100.0) | 0 | 0.8 (1.1) | 0.5 (0.9) | <0.001 |
|  | 8 | 317,852 (100.0) | 0 | 0.8 (1.1) | 0.5 (0.9) | <0.001 |
|  | 9 | 317,852 (100.0) | 0 | 0.7 (1.1) | 0.5 (0.9) | <0.001 |
|  | 10 | 317,852 (100.0) | 0 | 0.7 (1.1) | 0.4 (0.9) | <0.001 |
|  | 11 | 317,852 (100.0) | 0 | 0.7 (1.1) | 0.4 (0.8) | <0.001 |
|  | 12 | 317,852 (100.0) | 0 | 0.6 (1.0) | 0.4 (0.8) | <0.001 |
| **Mean hospital admission counts** | 1 | 317,852 (100.0) | 0 | 0.0 (0.1) | 0.0 (0.1) | <0.001 |
|  | 2 | 317,852 (100.0) | 0 | 0.0 (0.1) | 0.0 (0.1) | <0.001 |
|  | 3 | 317,852 (100.0) | 0 | 0.0 (0.1) | 0.0 (0.1) | <0.001 |
|  | 4 | 317,852 (100.0) | 0 | 0.0 (0.1) | 0.0 (0.1) | <0.001 |
|  | 5 | 317,852 (100.0) | 0 | 0.0 (0.1) | 0.0 (0.1) | <0.001 |
|  | 6 | 317,852 (100.0) | 0 | 0.0 (0.1) | 0.0 (0.1) | <0.001 |
|  | 7 | 317,852 (100.0) | 0 | 0.0 (0.1) | 0.0 (0.1) | <0.001 |
|  | 8 | 317,852 (100.0) | 0 | 0.0 (0.1) | 0.0 (0.1) | <0.001 |
|  | 9 | 317,852 (100.0) | 0 | 0.0 (0.1) | 0.0 (0.1) | <0.001 |
|  | 10 | 317,852 (100.0) | 0 | 0.0 (0.1) | 0.0 (0.1) | <0.001 |
|  | 11 | 317,852 (100.0) | 0 | 0.0 (0.1) | 0.0 (0.1) | <0.001 |
|  | 12 | 317,852 (100.0) | 0 | 0.0 (0.1) | 0.0 (0.1) | <0.001 |
| **Mean A&E visits** | 1 | 317,852 (100.0) | 0 | 0.1 (0.3) | 0.0 (0.2) | <0.001 |
|  | 2 | 317,852 (100.0) | 0 | 0.0 (0.2) | 0.0 (0.2) | <0.001 |
|  | 3 | 317,852 (100.0) | 0 | 0.0 (0.2) | 0.0 (0.2) | <0.001 |
|  | 4 | 317,852 (100.0) | 0 | 0.0 (0.2) | 0.0 (0.2) | <0.001 |
|  | 5 | 317,852 (100.0) | 0 | 0.0 (0.2) | 0.0 (0.2) | <0.001 |
|  | 6 | 317,852 (100.0) | 0 | 0.0 (0.2) | 0.0 (0.2) | <0.001 |
|  | 7 | 317,852 (100.0) | 0 | 0.0 (0.2) | 0.0 (0.2) | <0.001 |
|  | 8 | 317,852 (100.0) | 0 | 0.0 (0.2) | 0.0 (0.2) | <0.001 |
|  | 9 | 317,852 (100.0) | 0 | 0.0 (0.2) | 0.0 (0.2) | <0.001 |
|  | 10 | 317,852 (100.0) | 0 | 0.0 (0.2) | 0.0 (0.1) | <0.001 |
|  | 11 | 317,852 (100.0) | 0 | 0.0 (0.2) | 0.0 (0.1) | <0.001 |
|  | 12 | 317,852 (100.0) | 0 | 0.0 (0.2) | 0.0 (0.1) | <0.001 |
| **Mean outpatient clinic visits** | 1 | 317,852 (100.0) | 0 | 0.3 (0.8) | 0.2 (0.6) | <0.001 |
|  | 2 | 317,852 (100.0) | 0 | 0.3 (0.8) | 0.1 (0.6) | <0.001 |
|  | 3 | 317,852 (100.0) | 0 | 0.3 (0.8) | 0.1 (0.5) | <0.001 |
|  | 4 | 317,852 (100.0) | 0 | 0.3 (0.8) | 0.1 (0.6) | <0.001 |
|  | 5 | 317,852 (100.0) | 0 | 0.3 (0.8) | 0.1 (0.5) | <0.001 |
|  | 6 | 317,852 (100.0) | 0 | 0.3 (0.8) | 0.1 (0.5) | <0.001 |
|  | 7 | 317,852 (100.0) | 0 | 0.3 (0.7) | 0.1 (0.5) | <0.001 |
|  | 8 | 317,852 (100.0) | 0 | 0.3 (0.7) | 0.1 (0.5) | <0.001 |
|  | 9 | 317,852 (100.0) | 0 | 0.2 (0.7) | 0.1 (0.5) | <0.001 |
|  | 10 | 317,852 (100.0) | 0 | 0.2 (0.7) | 0.1 (0.5) | <0.001 |
|  | 11 | 317,852 (100.0) | 0 | 0.2 (0.7) | 0.1 (0.5) | <0.001 |
|  | 12 | 317,852 (100.0) | 0 | 0.2 (0.6) | 0.1 (0.4) | <0.001 |
| **Mean admission costs (£)** | 1 | 317,800 (100.0) | 55 | 2,577.4 (4523.6) | 3,258.2 (4802.9) | <0.001 |
|  | 2 | 317,805 (100.0) | 50 | 2,989.7 (4677.2) | 3,250.8 (4676.5) | 0.05 |
|  | 3 | 317,810 (100.0) | 45 | 3,011.3 (4694.7) | 3,317.4 (5108.1) | 0.04 |
|  | 4 | 317,805 (100.0) | 45 | 3,014.6 (4159.7) | 3,427.9 (4957.0) | 0.01 |
|  | 5 | 317,805 (100.0) | 50 | 3,194.0 (5317.6) | 3,331.4 (4959.2) | 0.38 |
|  | 6 | 317,815 (100.0) | 35 | 2,934.0 (4675.4) | 3,510.4 (5596.4) | <0.001 |
|  | 7 | 317,815 (100.0) | 35 | 3,016.5 (5191.6) | 3,437.0 (5191.7) | 0.01 |
|  | 8 | 317,805 (100.0) | 45 | 2,955.6 (4278.8) | 3,387.7 (4852.3) | 0.01 |
|  | 9 | 317,810 (100.0) | 45 | 3,212.0 (4534.4) | 3,451.1 (5153.9) | 0.15 |
|  | 10 | 317,820 (100.0) | 35 | 3,287.4 (6008.7) | 3,255.9 (5293.1) | 0.87 |
|  | 11 | 317,810 (100.0) | 40 | 2,993.0 (3993.4) | 3,115.6 (4485.7) | 0.43 |
|  | 12 | 317,820 (100.0) | 30 | 2,898.7 (4348.0) | 3,236.4 (5059.9) | 0.07 |
| **Mean A&E visit costs (£)** | 1 | 317,850 (100.0) | 0 | 177.3 (117.4) | 161.2 (114.4) | <0.001 |
|  | 2 | 317,850 (100.0) | 0 | 165.8 (115.3) | 160.4 (117.1) | 0.08 |
|  | 3 | 317,850 (100.0) | 0 | 168.9 (113.6) | 159.5 (115.0) | 0.00 |
|  | 4 | 317,850 (100.0) | 0 | 163.3 (103.4) | 161.3 (115.9) | 0.53 |
|  | 5 | 317,850 (100.0) | 0 | 164.5 (108.2) | 157.6 (115.2) | 0.03 |
|  | 6 | 317,850 (100.0) | 0 | 162.3 (102.8) | 159.0 (108.8) | 0.29 |
|  | 7 | 317,850 (100.0) | 0 | 158.3 (104.2) | 156.9 (104.3) | 0.65 |
|  | 8 | 317,850 (100.0) | 0 | 159.6 (103.8) | 159.3 (107.0) | 0.92 |
|  | 9 | 317,850 (100.0) | 0 | 162.0 (112.5) | 156.5 (108.2) | 0.11 |
|  | 10 | 317,850 (100.0) | 0 | 158.1 (109.2) | 157.8 (104.7) | 0.93 |
|  | 11 | 317,850 (100.0) | 0 | 164.9 (123.7) | 156.2 (114.8) | 0.03 |
|  | 12 | 317,850 (100.0) | 0 | 154.4 (106.2) | 155.9 (115.5) | 0.70 |
| **Mean outpatient clinic visit costs (£)** | 1 | 311,595 (98.0) | 6,260 | 89.0 (125.4) | 97.9 (139.6) | <0.001 |
|  | 2 | 311,720 (98.1) | 6,135 | 91.6 (126.7) | 94.9 (128.9) | 0.04 |
|  | 3 | 311,605 (98.0) | 6,250 | 91.9 (139.3) | 96.6 (129.0) | 0.00 |
|  | 4 | 311,845 (98.1) | 6,005 | 94.1 (125.9) | 97.9 (128.6) | 0.02 |
|  | 5 | 311,905 (98.1) | 5,950 | 97.5 (126.3) | 97.0 (125.9) | 0.73 |
|  | 6 | 312,020 (98.2) | 5,830 | 93.4 (129.6) | 97.3 (128.2) | 0.02 |
|  | 7 | 312,345 (98.3) | 5,510 | 94.4 (128.0) | 98.0 (127.2) | 0.04 |
|  | 8 | 312,435 (98.3) | 5,420 | 96.7 (132.9) | 97.0 (128.4) | 0.87 |
|  | 9 | 312,755 (98.4) | 5,100 | 93.3 (122.5) | 96.5 (127.8) | 0.07 |
|  | 10 | 313,145 (98.5) | 4,710 | 94.9 (129.6) | 98.2 (127.3) | 0.09 |
|  | 11 | 313,365 (98.6) | 4,490 | 94.5 (135.8) | 97.2 (125.8) | 0.17 |
|  | 12 | 313,860 (98.7) | 3,995 | 92.6 (127.3) | 97.3 (125.9) | 0.03 |
| **Mean follow-up time (days)** | 1 | 317,789 (100.0) | 63 | 29.7 (2.4) | 29.7 (2.4) | 0.52 |
|  | 2 | 311,081 (97.9) | 6,771 | 29.7 (2.3) | 29.7 (2.3) | 0.25 |
|  | 3 | 304,922 (95.9) | 12,930 | 29.6 (2.8) | 29.6 (2.7) | 0.70 |
|  | 4 | 296,704 (93.3) | 21,148 | 29.6 (2.8) | 29.6 (2.8) | 0.49 |
|  | 5 | 287,733 (90.5) | 30,119 | 29.6 (2.7) | 29.6 (2.7) | 0.50 |
|  | 6 | 279,906 (88.1) | 37,946 | 29.6 (2.9) | 29.6 (2.9) | 0.66 |
|  | 7 | 271,527 (85.4) | 46,325 | 29.4 (3.3) | 29.4 (3.4) | 0.23 |
|  | 8 | 260,126 (81.8) | 57,726 | 29.3 (3.6) | 29.3 (3.6) | 0.80 |
|  | 9 | 248,128 (78.1) | 69,724 | 29.4 (3.1) | 29.4 (3.1) | 0.83 |
|  | 10 | 237,322 (74.7) | 80,530 | 29.2 (3.9) | 29.2 (3.9) | 0.50 |
|  | 11 | 222,732 (70.1) | 95,120 | 28.7 (5.0) | 28.6 (5.0) | 0.73 |
|  | 12 | 202,073 (63.6) | 115,779 | 28.9 (4.4) | 28.9 (4.5) | 0.51 |
