# Supplementary material for: Healthcare utilisation in people with long COVID: an OpenSAFELY cohort study
Source: BMC Med. 2024 Jun 20;22:255. doi: 10.1186/s12916-024-03477-x (PMC11188519; doi:10.1186/s12916-024-03477-x)
Supplement: Supplementary file 7 — Additional file 7. [file 12916_2024_3477_MOESM7_ESM.docx]

**Table S6.** Distribution of Healthcare Visits and Person-Time Across Different Outcomes for Long COVID Exposure and Comparator Groups

| **Outcomes** | **Exposure** | **Numbers** | **Healthcare visit counts** | **Person-time (days)** |
| --- | --- | --- | --- | --- |
| Total healthcare utilizations | Comparator | 175,015 | 2,812,215 | 53,676,700 |
|  | Long covid exposure | 42,545 | 1,041,585 | 12,765,255 |
| GP visits | Comparator | 158,590 | 1,079,215 | 49,507,955 |
|  | Long covid exposure | 40,030 | 428,860 | 12,081,215 |
| Prescription visits | Comparator | 147,805 | 1,334,875 | 45,373,425 |
|  | Long covid exposure | 38,750 | 441,065 | 11,717,720 |
| Hospital admission | Comparator | 10,780 | 14,775 | 3,400,180 |
|  | Long covid exposure | 3,410 | 4,885 | 1,071,680 |
| A&E visits | Comparator | 32,735 | 49,025 | 10,589,925 |
|  | Long covid exposure | 11,075 | 18,285 | 3,561,310 |
| Outpatient clinic visits | Comparator | 75,650 | 334,325 | 23,891,150 |
|  | Long covid exposure | 26,745 | 148,485 | 8,271,340 |
