# Supplementary material for: Healthcare utilisation in people with long COVID: an OpenSAFELY cohort study
Source: BMC Med. 2024 Jun 20;22:255. doi: 10.1186/s12916-024-03477-x (PMC11188519; doi:10.1186/s12916-024-03477-x)
Supplement: Supplementary file 10 — Additional file 10. [file 12916_2024_3477_MOESM10_ESM.docx]

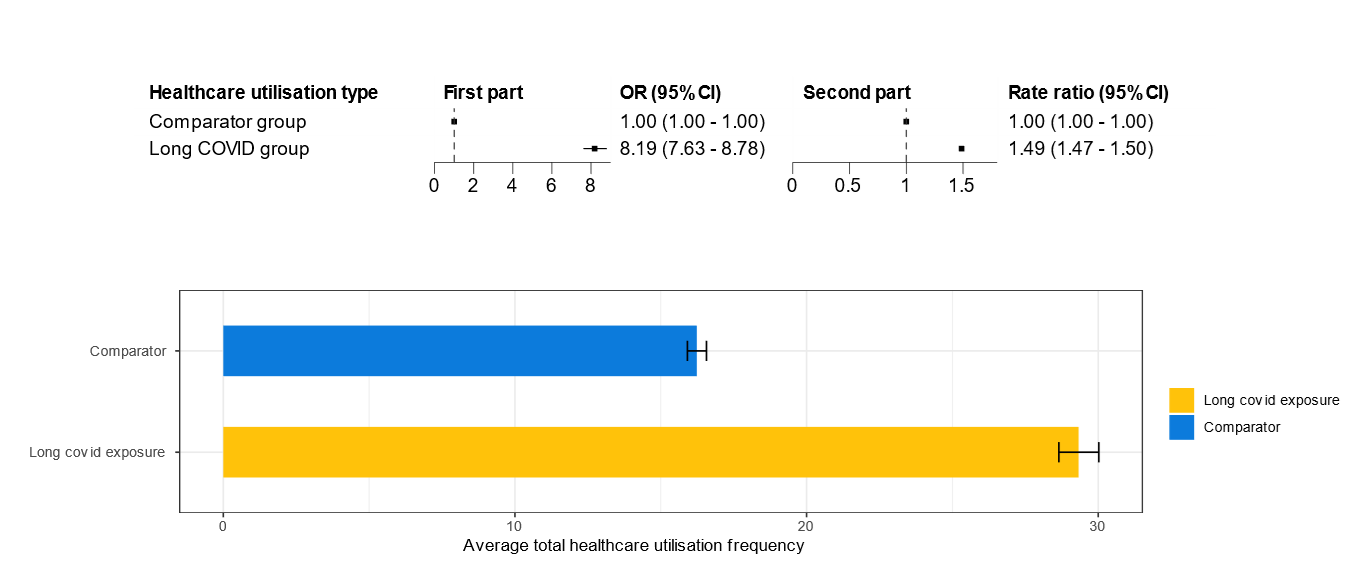


**Fig. S7.** Sensitivity analyses among people who had been registered to a GP and visited a GP one year before the study follow-up
