# Supplementary material for: Healthcare utilisation in people with long COVID: an OpenSAFELY cohort study
Source: BMC Med. 2024 Jun 20;22:255. doi: 10.1186/s12916-024-03477-x (PMC11188519; doi:10.1186/s12916-024-03477-x)
Supplement: Supplementary file 13 — Additional file 13. [file 12916_2024_3477_MOESM13_ESM.docx]

### Table S1. Long COVID SNOMED codes

| **SNOMED codes** | **Term** |
| --- | --- |
| 1325161000000100 | Post-COVID-19 syndrome |
| 1325181000000100 | Ongoing symptomatic disease caused by severe acute respiratory syndrome coronavirus 2 |
| 1325021000000100 | Signposting to Your COVID Recovery |
| 1325031000000100 | Referral to post-COVID assessment clinic |
| 1325041000000100 | Referral to Your COVID Recovery rehabilitation platform |
| 1325051000000100 | Newcastle post-COVID syndrome Follow-up Screening Questionnaire |
| 1325061000000100 | Assessment using Newcastle post-COVID syndrome Follow-up Screening Questionnaire |
| 1325071000000100 | COVID-19 Yorkshire Rehabilitation Screening tool |
| 1325081000000100 | Assessment using COVID-19 Yorkshire Rehabilitation Screening tool |
| 1325091000000100 | Post-COVID-19 Functional Status Scale patient self-report |
| 1325101000000100 | Assessment using Post-COVID-19 Functional Status Scale patient self-report |
| 1325121000000100 | Post-COVID-19 Functional Status Scale patient self-report final scale grade |
| 1325131000000100 | Post-COVID-19 Functional Status Scale structured interview final scale grade |
| 1325141000000100 | Assessment using Post-COVID-19 Functional Status Scale structured interview |
| 1325151000000100 | Post-COVID-19 Functional Status Scale structured interview |
