# Supplementary material for: Healthcare utilisation in people with long COVID: an OpenSAFELY cohort study
Source: BMC Med. 2024 Jun 20;22:255. doi: 10.1186/s12916-024-03477-x (PMC11188519; doi:10.1186/s12916-024-03477-x)
Supplement: Supplementary file 15 — Additional file 15. [file 12916_2024_3477_MOESM15_ESM.docx]

### Table S2. List of comorbidities

| **Disease** |
| --- |
| Non-haematological cancer |
| Haematological cancer^1^ |
| Chronic respiratory disease |
| Chronic cardiac disease |
| Chronic liver disease |
| Stroke or dementia |
| Other neurological conditions^2^ |
| Organ transplant |
| Rheumatoid arthritis |
| Systemic lupus erythematosus |
| Psoriasis |
| Other immunosuppressive conditions^3^ |

1. Having haematological cancers six months before the index date; 2. Such as Huntington’s disease, multiple sclerosis, motor neuron diseases, and other neurological diseases; 3. Including other permanent and temporary immunosuppressive diseases.
